# Automatic Differentiation for Inverse Problems in X-ray Imaging and Microscopy

**DOI:** 10.3390/life13030629

**Published:** 2023-02-23

**Authors:** Francesco Guzzi, Alessandra Gianoncelli, Fulvio Billè, Sergio Carrato, George Kourousias

**Affiliations:** 1 Elettra—Sincrotrone Trieste, Strada Statale 14—km 163,500 in AREA Science Park, Basovizza, 34149 Trieste, Italy; 2 Department of Engineering and Architecture (DIA), University of Trieste, 34127 Trieste, Italy

**Keywords:** soft-X-ray microscopy, automatic differentiation, computational imaging, parameter refining, inverse problems

## Abstract

Computational techniques allow breaking the limits of traditional imaging methods, such as time restrictions, resolution, and optics flaws. While simple computational methods can be enough for highly controlled microscope setups or just for previews, an increased level of complexity is instead required for advanced setups, acquisition modalities or where uncertainty is high; the need for complex computational methods clashes with rapid design and execution. In all these cases, Automatic Differentiation, one of the subtopics of Artificial Intelligence, may offer a functional solution, but only if a GPU implementation is available. In this paper, we show how a framework built to solve just one optimisation problem can be employed for many different X-ray imaging inverse problems.

## 1. Introduction

Inverse problems such as nanotomography [1], compressive sensing [2], super-resolution [3] and ptychography [4] are solved by exploiting the relation between data simulated through a model and what is detected during the measurements. Common iterative reconstruction algorithms make use of gradient-based optimisation to reconstruct the specimen *x*, which is the solution to the system of equations governing the model [5]. These approaches are powerful but can be problematic [6,7]: if a sophisticated method can simulate realistic behaviours, adding complexity results in error-prone expressions to be derived manually; this is especially true when the parameters must also be refined (e.g., in tomography [8,9]). If the object unknowns lie in a transform domain (e.g., wavelets), the complexity is also further increased. Pushed by Artificial Intelligence and machine learning research, Automatic Differentiation (AD) [10,11,12] methods represent an alternative in computational imaging, as they provide the key to prototypes in a simple manner for state-of-the-art reconstruction methods. Apart from being mathematically correct and powerful, resulting reconstruction algorithms are also fast if GPU/parallel implementations can be readily coded.

### 1.1. Paper Scope

The main scope of this manuscript is to showcase how AD-based methods can be used to open the black box of many computational imaging problems. Following our framework, it is possible to rapidly prototype state-of-the-art solutions for computational microscopy methods. Possible examples are: a compressive sensing reconstruction algorithm for sparse datasets, a single image super-resolution algorithm for recovering missing information from a low-resolution image, a 2D/3D tomography reconstruction algorithm that can deal with the missing wedge problem, sparse acquisition and axial misalignment, and a ptychography algorithm, which can retrieve many geometry parameters [13]. Even if the aforementioned solutions are purely “conventional”, the use of such an AI component as AD blurs out the distinction between classical and AI methods [12,14,15].

In Section 1.2, we introduce the concept of Automatic Differentiation; then, in Section 1.3, we describe the generic computational imaging problem statement. We also provide an introduction to the four imaging techniques discussed in this manuscript (that can eventually be skipped by experienced readers). In the method section, we describe the proposed AD-based computational framework, and finally, present the results that can be obtained.

### 1.2. Potential Uses of Automatic Differentiation

Calculating derivatives is a need for many numerical techniques that require numerical optimisation. While gradient-less methods [16] are used to explore solution spaces of a few dimensions (e.g., Bayesian methods to find hyper-parameters), gradients are essential in minimisation problems that involve a huge number of parameters, such as the training of neural networks [11,14,17] or a reconstruction of a computational imaging framework. Any use of finite difference methods would result in a very long process. On the other side, as the computational pipelines and the models become more complicated, computing gradients by hand becomes a challenging, time-consuming, difficult and error-prone task [12]. For many problems, computing derivatives analytically is simply impossible, and only an evaluation at certain points is feasible [12].

*Hand-coded analytical gradients* provide an exact expression (when available) but can be time-consuming to code properly (e.g., on hardware accelerators), error-prone, and not applicable to problems with implicit solutions [10,11].

*Finite differentiation*, on the other hand, is extremely easy to implement but is subject to fixed numerical precision and is slow, as the method requires one evaluation for each dimension [12]. This immediately makes it infeasible for many tasks involving direct image optimisation.

*Symbolic differentiation* addresses the weaknesses of both the manual and numerical methods but often results in complex and cryptic expressions plagued with the problem of “expression swell” [11], referring to the phenomenon of a much larger representation of the derivative as opposed to the representation of the original function [18]. In addition to this, symbolic differentiation tends to also be memory intensive, slow and cannot handle common statements (e.g., unbounded loops) [12].

*Automatic Differentiation* is a set of techniques aiming at evaluating the derivative of a mathematical function specified in a code. AD interprets the program by incorporating derivative values at each node of a *computational graph* and propagates their values following the chain rule of differential calculus [10]. In a differentiable programming language such as PyTorch [19], indeed, one can write a differentiable expression acting on a multi-dimensional numerical array, and a computational graph is built node-by-node as the temporary results are calculated. When the gradient of the leaf with respect to a particular node is requested, the computational graph is followed by reverse-concatenating the sequence of known partial derivatives, following the chain rule [10,19]. This constitutes the additional computational step that makes AD methods slower than a well-coded analytical gradient, which can be immediately calculated from a hand-typed and potentially well-simplified expression. However, this problem can be consistently alleviated if GPU-accelerated frameworks such as PyTorch are used.

### 1.3. Computational Imaging Problem Statement

Inverse problems are frequently solved through iterative procedures that refine the estimate of a desired latent quantity *x*; this refinement can be formalised as the minimisation of an error function L(x), which measures how dissimilar the simulations $\hat{y}$ are with respect to the acquired data points *y*. These simulations are produced by applying to the latent quantity *x* an operator A describing the computational model of the particular technique at hand. Intuitively, a good estimate of *x* thus permits to simulate quantities that closely follow the measured dataset.

The complete loss expression for a generic image inverse problem is shown in Equation (1)

(1) $$L\left(x\right)=||Ax-{y||}_{2}^{2}+{\left||x|\right|}_{1}=\left|\right|\hat{y}-{y||}_{2}^{2}+{\left||x|\right|}_{1}.$$

The Limited-memory-Broyden–Fletcher–Goldfarb–Shanno algorithm (L-BFGS) [20] or the Orthant-Wise Limited-memory Quasi-Newton method (OWL-QN) [21] are commonly used solvers, but a gradient expression is required. While for simple expressions one can manually calculate it, such as in Equation (2) (to refine *x*) and in Equation (3) (to refine A)

(2) $$\nabla_{x}L\left(x\right)=2A^{T}\left(Ax-y\right)+\frac{x}{\left|x\right|}$$

(3) $$\nabla_{A}L\left(x;A\right)=2\left(Ax-y\right)\;\cdot \;x^{T},$$

for cumbersome models (especially if complex-valued quantities are involved), AD methods come in handy, providing exact gradient expressions for the actual A,x,y values [10]. The only requirement is to write a differentiable expression as a function of the tensors that should be optimised.

### 1.4. Compressive Sensing

Compressive sensing (CS) [2,22,23] refers to a particular sampling modality that explicitly violates the Nyquist rate. Any discrete signal *x* of dimension *N* can be expressed as a linear combination of *N* basis vectors. In many practical cases, the signal *x* is not sparse in the sampling domain, but it can be considered *K-sparse* (K≪N coefficients are nonzero) if another set of basis vectors, e.g., from Discrete Cosine Transform (DCT) or Discrete Wavelet Transform (DWT), is chosen. When this condition is met, the signal is also *compressible* [22], and one can effectively reconstruct the whole latent image, even from the set of sparse measurements in the sampling domain [2].

During Scanning Transmission X-ray Microscopy (STXM) or X-ray Fluorescence (XRF) experiments [24], CS has been employed to dramatically reduce the time acquired for a single scan [25,26]: based on a rough STXM measurement, indeed, a spatial mask can be used to discriminate areas of sparse or fine scanning (for the subsequent XRF/STXM fine-measure). A different application is in Fourier Holography, where a CS framework is employed to solve for the values of the diffraction pattern, which are covered by a beam-stop at the detector plane [27].

The computational model for a CS reconstruction can be formalised exactly as in Equation (1), where A is substituted by the matrix product ΦΨ as in the following expression:

(4) $$L\left(x\right)=||\Phi \Psi x-{y||}_{2}^{2}+{\left||x|\right|}_{1};$$

Ψ denotes the 2D inverse cosine or wavelet transform operator applied on the transform coefficients vector *x*; in this latent space *x*, one can take into account energy considerations by leveraging the L1 regularisation. To reconstruct the latent image Ψx, the minimisation problems of Equation (4) have to be set. Finally, Φ represents the sparse sampling operation, where only a fraction of the image pixels is measured.

The taxonomy [28,29] of the CS reconstruction methods (all iterative) involves typically three classes of algorithms: (1) iterative-thresholding algorithms based on Douglas–Rachford (DR) splitting [30,31]; (2) greedy algorithms such as Orthogonal Matching Pursuit (OMP) [32,33,34]; (3) gradient-based optimisations based on Equation (1).

When comparing these algorithms, gradient-based optimisation provides the best reconstruction accuracy, but at the cost of very high computational complexity. Greedy algorithms can be fast and very accurate but tend to stagnate for high-dimensional space and regular sampling, while the thresholding algorithms are fast and frequently used due to their simplicity but can provide severely blurred results [28].

Image *inpainting* is another common way to “fill the blanks” in the measured image [35]; This kind of method essentially solves a Partial Differential Equation describing how grey levels propagate inside missing regions [36], by continuing the lines of equal grey value *(isophotes)*; in [35], a corrupted image is restored by solving the 2D Poisson Equation employing biharmonic functions; the Telea algorithm [36] aims at producing a very fast interpolation by taking into account a slow-varying “baseline” and the estimated value of the 2D gradient. The Naivier–Stokes (NS) method [37] works by assessing many similarities between 2D image quantities (intensity, isophote direction, smoothness, anisotropic diffusion) and fluid-related ones (stream function, fluid velocity, vorticity, fluid viscosity). Frequency Selective Reconstruction (FSR) [38] is a block-based algorithm that approximates the known samples by a weighted superposition of Fourier basis functions to estimate the lost pixels [39]; in some ways, it follows the reconstruction paradigm in CS.

### 1.5. Single Image Super Resolution

Single Image Super Resolution (SISR) [3] is a class of computational methods that aims at retrieving a High Resolution (HR) image from a single degraded Low Resolution (LR) one; indeed, finer details that are typically within the size of the former spatial sampling period can eventually be made visible.

One of the most used degradation models [40] is:

(5) y=BHx+n

where *y* is the degraded LR frame, *x* is, as usual, the latent HR image, H is the blur operator, B is the down-sampling operator and *n* the noise. While the traditional super-resolution approach employs multiple frames to recover the missing information (e.g., in order to obtain a readable license plate image from a surveillance video [41]), the SISR inverse problem is extremely *ill-posed* as many more different HR images can be projected to the same LR one [42]. This issue clearly emerges when simple interpolation methods are used, leading to the blurring or the generation of artefacts around edges and corners [43].

The literature on SISR evolved (a recent review is in [40]) mainly in four different branches: *(i) reconstruction-based methods* [44] are the oldest ones and aim at recovering the latent HR image by approximating the degradation process via a composition of blurring, downsampling, and noise injection; the method in [45] solves a Maximum Likelihood optimisation problem by employing a set of well-behaving image priors for the noise and image distribution. Turbozoom [46] is a recent reconstruction-based algorithm that employs a fast Conjugate Gradient optimisation to solve the degradation problem in Equation (5). However, in the majority of works, many solutions adopt a simpler degradation model, e.g., directly downscaling an HR image using a gaussian or a bicubic kernel to generate the corresponding LR one [40].

*(ii) Sparse-based algorithms* [47] are another class of reconstruction methods where convergence is improved by exploiting the sparsity characteristics in the transform domain of the latent image, similar to a CS problem. One notable example is [48] where a CS framework is adapted for super-resolution by introducing a fixed blur kernel. The complete degradation model is thus composed of a blur operator and regular sparse scanning (sub-sampling) in the spatial domain. The regular sampling produces a structured mask, defining a critical condition for CS. By introducing the blur kernel, the (Restricted Isometry property) RIP condition is again met, making an OMP algorithm converge again.

*(iii) Example/patch-based algorithms* [40] historically provided the best results from a purely aesthetic point of view [49]; the inner working relies on generating a self-dictionary of high-resolution patches, which can be used to generate an HR frame [50]. As their use is questionable in the scientific, clinical or forensic cases, these kinds of algorithms are not considered in this manuscript.

SISR is currently receiving lots of attention from both the industry and the Image Processing research community, especially after the advent of *(iv) Deep-Learning-based methods*, which produce so-called “hallucinations” [40,42]: during a learning phase, a trainable model learns to estimate the most probable HR patch. Recent notable examples of these kinds of state-of-the-art methods involve the use of Convolutional Neural Networks (CNN), such as ESDR [51], ESPCN [52], FSRCNN [53], LAPSRN [54]) or Noise2Noise [42]; this is similar to the case of example-based SISR techniques where for natural images, the aesthetic factor can be privileged; in microscopy, instead, we need to assure that the reconstructed image holds high fidelity with the ground truth (the sample). The same reasoning can be applied to CNNs, if we are uncertain about their inner working [55], as the *hallucinated* images are strongly biased by the nature of the training dataset. As an example, the model used in [42] is provided pre-trained for different types of datasets, e.g., faces and walls, even if the task for what the model is trained is the same. The field of explainable AI [56] is growing in response to these assumptions [57], creating both less-opaque models and techniques to inspect the decisional process of AI [56,58].

### 1.6. Tomography

X-ray Computer tomography (CT) [59,60] is used to analyse biological samples in their native environment [61] in a non-destructive way. In its simplest form [62], we aim at reconstructing a 3D volume starting from a series of 2D projections $y_{i}$, acquired at different rotation angles $\theta_{i}$, where *i* denotes the *i*th observation [1]. This inverse problem is formally still another formulation of Equation (1), where A is substituted with $MR_{\theta_{i}}$, such as in Equation (6)

(6) $$L\left(x\right)=\sum_{i}||MR_{\theta_{i}}x-y_{i}{\left|\right|}_{2}^{2}.$$

The latent 3D object *x* is illuminated by a high-dose parallel field and is rotated on a vertical axis by the operator $R_{\theta_{i}}$; the rotation axis lays exactly in the middle of the object, its projection is in the middle of the detector and is perpendicular to the beam. A Riemann sum operator M describes the ray-tracing within the sample, following the projection approximation [63] and the Lambert–Beer law [64]. The Crowther criterion [65] establishes the maximum angle step size that is required to fully recover the Fourier space of the sample. While the Filtered Back Projection (FBP)/gridrec [59,66] and the Algebraic Reconstruction (ART) [67] provides satisfactory reconstructions, in many micro/nano-CT experiments different technical difficulties can arise (here are just a few examples):

(i) Radiation damage sets limits on the beam intensity for each projection [68]; when the number of photons is scarce (e.g., due to dose fractionation criteria [69] or photon starvation [70]), common single-shot algorithms produce very noisy images [60]. Ex-post noise removal increases the output Signal-to-Noise-Ratio (SNR) at the expense of spatial resolution. A meaningful reconstruction can be obtained by employing a Maximum-Likelihood algorithm (e.g., with Expectation-Maximisation (MLEM) [71]), which exploits a priori information on the noise process.

(ii) Satisfying the Crowther criterion might not always be possible, e.g., due to time/dose restrictions as well as mechanical limitations; iterative algorithms [64], such as SIRT [72], PSIRT [73], or MLEM [71], make use of the same concepts of regularisation and sparsity discussed so far to restrict the solution space to likely values.

(iii) In micro/nano-CT, various sample configurations limit the range of accessible tilt angles to 100–120${}^{\circ }$ (missing wedge [74]) due to crowded experimental chambers (e.g., cryo stage in cryo-nano-tomography [61,75]), nanopositioning stage limits or large absorption in the sample; while single-shot algorithms would produce severely corrupted reconstructions, iterative algorithms exploiting regularisation, and a CS approach [76] can at least provide good spatial resolution in a plane parallel to the detector [74,77].

The problems discussed so far produce very noticeable and characteristic artefacts in the reconstructions: while FBP output images are noisy, have poor contrast and are blurry [60], iterative methods might generate sub-optimal image texture, often referred to as “plastic” or “blotchy” [78]. In this context, AI can provide a huge help, as it can learn the correspondence between the latent object and the characteristic artefact/defect triggered in a well-defined reconstruction algorithm by a well-defined problem [79]. A review of methods is in [60].

In cryo-nanotomography [75,80], the aforementioned problems appear at the same time and are also exacerbated by unwanted and unknown movements in the sample stage [9,81,82]. A simple correction model employs serial cross-correlation between projections [83], also with cosine-stretching, reducing the risk of potentially propagating drifts in the correction [84]. Even if, in some cases, an algorithm might be able to create a set of landmarks from image features alone [85,86,87,88], marker-based alignment methods [84,89,90,91,92,93] provide the de-facto solution to the problem; at the cost of decreasing the sample visibility, gold-nanobeads are added to the sample solutions, providing a set of landmarks that are relatively easy to detect and track automatically (but manual tracking is often required for a variety of reasons [75,94]). Stretched cross-correlation is still used as a pre-alignment step. A different class of algorithms is formalised around the concept of *tomography self-consistency*, which tries to infer the alignment parameters while reconstructing the volume. These methods can exploit both gradient-less [81,94] or gradient-based optimisation [95,96] to infer the parameters of a model significantly more complex than the one in Equation (6).

### 1.7. Ptychography

Ptychography [4,97] aims to dramatically increase the space-bandwidth product of a microscopy experiment by exploiting a computational imaging approach [98]. In a transmission setup, an object is raster scanned with a spatially coherent, monochromatic and time-stationary illumination *p*; the field is a spherical wave emerging from a pinhole or a Fresnel Zone Plate (FZP) [4]. In the thin-sample approximation [99], the input field is simply modulated in magnitude and phase by the object transmission function *x*. A 2D detector, typically placed in the far-field [4] at a distance *z*, records the diffraction pattern $|d_{i}{|}^{2}$ of the transmitted field; the use of this regime is used to ease the computational part, as the computational propagation $D_{z}$ can be approximated with a 2D Fourier transform [4]. If only one diffraction pattern is collected, this approach corresponds to Keyhole-CDI [100], but when multiple diffraction patterns are acquired in an over-scan fashion, the overlap between different beam positions provides the *diversity* [98], which is required to over-constrain the computational problem [98], described by Equation (7)

(7) $$|d_{i}{|}^{2}={\left|D_{z}\left\{p\;\cdot \;x_{i}\right\}\right|}^{2}$$

where, differently from the previous cases, $d_{i}$, *p* and $x_{i}$ are complex-valued quantities. The approach allows extending the Field Of View (FOV) of the acquired area ideally without limits, as long as a good overlap is maintained, paired with constancy in the illumination conditions. While the first proposed algorithm was a single-shot process [101], in conventional experiments, iterative algorithms based on the concepts of “projection” are extensively used: Ptychography Iterative Engine (PIE) [4], extended-PIE (ePIE) [102], Refined-PIE (rPIE) [103] and Alternating Projection (AP) [104] employ a sequential approach that is considered essential to exploring the solution space as much as possible; the Differential Map (DM) algorithm [105] instead makes an averaged update taking into account all the experiment data. A different approach based on a form of Equation (1) was also proposed [6] but initially considered too slow compared to projection-based methods. Apart from PIE, thanks to the diversity in the dataset, all the aforementioned algorithms can automatically solve for an estimate of the illumination field, similar to a blind-deconvolution process. The complications, which can be accounted for by extending the simple ptychography model of Equation (7), closely follow what has been described for tomography (here, just a few examples):

(i) In the case of a diffraction pattern with low SNR, knowledge on the noise statistics is essential; a Maximum Likelihood approach based on Gaussian or Poissonian noise has been described in [7], also with regularisation [106];

(ii) A trade-off between flux and spatial coherence of the beam is often required. When multiple propagating modes interfere, the resulting diffraction pattern appears blurred [107]. Based on the decomposition of the mutual intensity function [108], probe decomposition techniques such as [109,110,111,112] are often employed to computationally disentangle multiple modes.

(iii) Mechanical precision of the sample stage limits the spatial resolution of the reconstruction. Being a scanning technique, ptychography relies on accurate knowledge of the sample position (at the pixel level) for each acquired diffraction pattern $|d_{i}{|}^{2}$. On many ptychography beamlines, an ex-post approach employing computational position refinement algorithms is inevitable. Many methods have been proposed, based both on a gradient-less, e.g., [113,114,115,116,117,118], or gradient-based approach, e.g., [6,13,119,120]

(iv) Ptychography relies on many other model parameters such as propagation distance, beam defocus and beam energy that allows correctly describing the experiment at hand. In [121], beam defocus is corrected through a complex genetic-algorithm optimisation; in [122], the authors demonstrate how position correction can mitigate beam energy and propagation distance errors, while in [123], a variant of the PIE algorithm is proposed (z-PIE) that automatically refines the propagation distance.

Similarly to the previous case, AI can be used to correct or to provide a good initialisation for a subsequent common iterative algorithm [124].

## 2. Materials and Methods

In this work, we show how an AD framework can be employed to rapidly design state-of-art solutions to computational imaging problems, starting from a set of acquired data *y*.

### 2.1. Compressive Sensing

In Section 1, we have seen how Equation (1) is adapted to model Compressive Sensing, resulting in Equation (4). A typical Python implementation of such gradient-base optimisation is shown in Listing 1, where Numpy [125] and Scipy [126] Python libraries are used for numerical computing. An optimiser object is initialised by providing the initial values of the latent array *x*, containing the transform values in the space of the 2D DCT. As can be seen, both a loss function and a hand-typed gradient expression are required (see line 20 of Listing 1). The loss comprises both a data fidelity term (the L2 norm of the difference y−ΦΨx) and a regularisation term (L1 norm of the transform coefficients *x*), which are defined by using the *functional representation* of the matrix operators Ψ and Φ; these matrix operators are indeed substituted by *functions* (2D IDCT [127] and an element-wise multiplication). The use of the gradient-less algorithms specified in the *method* parameter in line 21 of Listing 1 would be impracticable. Note that this process is carried out on the CPU—single core—even if multiple CPUs are available. Porting this simple code on GPU is a non-trivial task.

**Listing 1.** Example implementation for Compressive Sensing gradient-based optimisation.

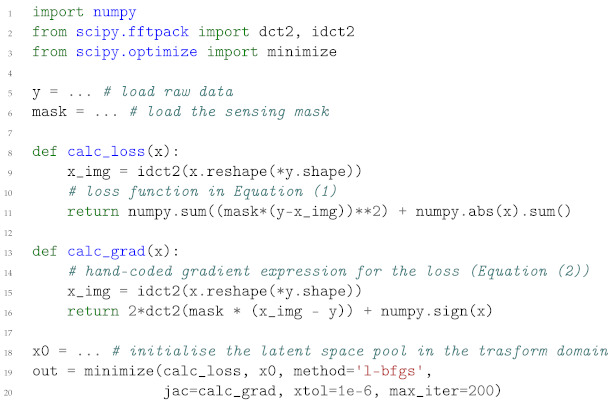



The equivalent code in a GPU-accelerated environment such as PyTorch is reported in Listing 2, where no gradient expression at all has to be manually calculated and coded.

**Listing 2.** AD implementation for the CS problem.

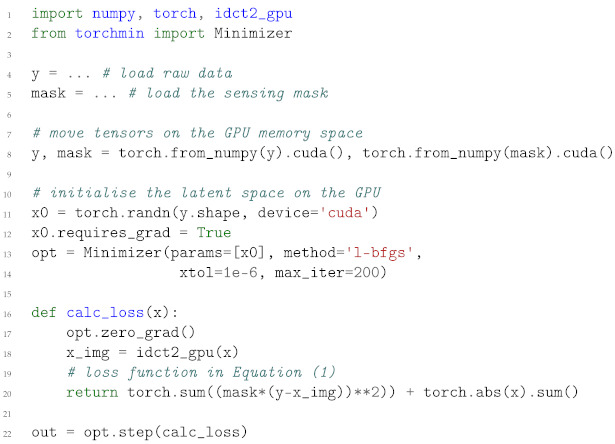



The loss function is defined as a *closure function* for the optimiser: in that function, the user describes all the steps required to produce the computational graph of the variable that has to be optimised (see *params* list in line 13 of Listing 2). At any iteration, the optimiser will (i) clear out the old gradient values; (ii) simulate the experiment in the forward pass; (iii) calculate the gradients by unrolling the computational graph from the loss value to the tensor *x*; (iv) calculate and apply a suitable update step for the variable in the list of *params*, following the particular algorithm at hand. The entire process is computed on the GPU with no memory transfers in the loop, as the functions (DCT transform [127] and generic float-array algebra) are defined and written to be executed through CUDA kernels and all the arrays have been moved to the GPU memory *before* the start of the optimisation loop (see line 8 and 12 in Listing 2). The resulting program is even faster than the previous single-threaded code, notwithstanding the hand-coded gradient in listing 1. While the optimiser itself is part of PyTorch [19], the interface used—*pytorch-minimise* [128]—allows writing a code, which is very familiar to experienced Numpy [125] users.

### 2.2. Single Image Super Resolution

The general degradation model in Equation (5) for Single Image Super Resolution can be formalised as an optimisation process similar to Equation (1), such as in Equation (8):

(8) $$L\left(x\right)=||\mathrm{BH}x-{y||}_{2}^{2}$$

where *x* is the unknown HR image, H is a 2D convolution operator that models the blur of the system with a convolution kernel *h*, and finally, B represents a warp operator (downscale) modelling the pixel size increase. As reported in Section 1.5, this model describes the low-resolution image as the output of blur with an unknown convolution kernel (13 × 13 in our experiments) and a fixed downscale (x4). Note that without operator B, Equation (8) is a blind deconvolution problem [129]. In [48], the blur and warp filter are modelled by only one filter, which is considered known (a gaussian with a width defined by the up-sample factor [46,129]); this represents a limiting factor.

In our SISR algorithm, we optmise both the HR image *x and* the deconvolution kernel *H*. To improve the quality of the result and the convergence, instead of the simple L2 norm $\left|\right|\hat{y}-{y|||}_{2}^{2}$, we used the Huber loss function [130], which is typically employed in Deep Learning problems:

(9) $$L_{\delta }\left(\hat{y},y\right)=\left\{\begin{array}{cc} \frac{1}{2}{\left(y-\hat{y}\right)}^{2} & if\left|(y-\hat{y})\right|<\delta \\ \delta (|y-\hat{y}|-\frac{1}{2}\delta ) & otherwise \end{array}\right.$$

where $\hat{y}=\mathrm{BH}x$. This loss function allows to speed up the convergence when the loss value is smaller than a parameter δ, which, in our case, is set to 1. This process-guided switch between the L1 and the L2 norm would be very difficult to implement in a conventional optimisation procedure. As in this case we are directly optimising the values of the HR image, a good choice for the regularisation term is the Total Variation [131]V(x), defined by Equation (10):

(10) $$V\left(x\right)=\sum_{i,j}\left(\right|x_{i+1,j}-x_{i,j}{|}^{2}+|x_{i,j+1}+x_{i,j}{|}^{2})$$

which acts by reducing the variations along adjacent pixels for any row *i* and any column *j*. By favouring slowly varying borders, we avoid the generation of high-frequency artefacts. The amount of regularisation in the loss function is controlled by the hyperparameters $\lambda_{1}$ and $\lambda_{2}$. The total loss function used to optimise the HR image *x* and blur kernel *h* values is thus:

(11) $$L\left(x,h\right)=L_{\delta }\left(\mathrm{BH}x,y\right)+\lambda_{1}V\left(x\right)+\lambda_{2}{\left||h|\right|}_{1}$$

which is implemented following the same reasoning in Listing 2.

### 2.3. Tomography

Even if the computational problem for CT is formally another formulation of Equation (1), for a real AD implementation the involved expressions start having a considerable complexity. For a parallel beam setup, the A matrix is indeed the composition of many operators, as shown in Equation (12):

(12) $$L\left(x;y\right)=\sum_{i}||MR_{\theta i}\Psi x-{y||}_{2}^{2}+\lambda_{1}{\left||x|\right|}_{1}+\lambda_{2}V\left(\Psi x\right)$$

The main component is still the L2 norm of the difference between each simulation and the acquired data. As discussed in Section 1.6, a minimisation in the space of the transform coefficients *x* is required, especially in the case of peculiar sampling conditions (e.g., limited angle and/or sparse acquisitions) [75]; by applying the Ψ operator on each slice, the full 3D volume is obtained. $R_{\theta i}$ acts on the 3D volume and rotates it along a vertical axis. Finally, M describes the Riemann sum of the sample slice, simulating the X-ray matter interactions within the pure projection approximation [1]. The regularisation terms are weighted by the hyper-parameters $\lambda_{1}$ and $\lambda_{2}$. In our implementation, similarly to the previous case, the Ψ matrix operator is substituted by a 2D IDWT [132] (Bi-orthogonal 4.4 [133]); $R_{i}$ employs an affine transform [13,17,134] to describe the rotation of $\theta_{i}$ degrees along an axis of parameterised coordinates $\left(r_{x},r_{y}\right)$. The Riemann sum is approximated with a sum along the propagation axis. All the functions are implemented in PyTorch and are GPU accelerated.

While the model described so far is sufficient for datasets that are dose-limited or with missing projections, it does not allow for geometrical parameter correction. One way to partially address the projection misalignment [81,94] described in Section 1.6 is to search for a set of detector shifts and a common detector tilt angle. To do this in AD, one can implement the model in Equation (13):

(13) $$L\left(x;y\right)=\sum_{i}||MR_{\theta i}\Psi x-C_{i}{y||}_{2}^{2}+\lambda_{1}{\left||x|\right|}_{1}+\lambda_{2}V\left(\Psi x\right)$$

where the operator $C_{i}$ applies an affine transform, which describes for each ith radiography a shift in x,y and a rotation that is global for the entire dataset. This operator is coded as $R_{\theta i}$. The total number of parameters for a dataset of *N* projections is indeed a 3D array containing the volume, 2N floats defying the shifts and an additional float for the common detector angle.

### 2.4. Ptychography

By raster-scanning the sample with a constant illumination *p* and by recording the corresponding *i*th diffraction pattern, one can reconstruct the complex refraction index of the total illuminated area of the sample. To do so, a computational method is required, as the missed phase for each diffraction pattern has to be retrieved. The model for this acquisition modality is even more intricate than the one in Equation (12) and is shown in Equation (14):

(14) $$L\left(x;p,z,r_{xi},r_{yi}\right)=\sum_{i}\left|\right|\sum_{m}|D_{z}p_{m}C_{i}{x|}^{2}-{y||}_{2}^{2}+\lambda_{1}\left|\right|x_{x>1}{\left|\right|}_{1},$$

where, again, the main loss component (what drives the reconstruction) is the sum of the L2 norms calculated for the difference between the simulations and the acquired data for each diffraction pattern. In this case, however, *x* is a complex-valued matrix describing the entire sample transmission function (entire Field Of View), $p_{m}$ is the multi-mode [109] complex valued illumination [109], $C_{i}$ is a cropping operator, which models the spatial scanning at the *i*th position $r_{xi},r_{yi}$[13]. Finally, $D_{z}$ is an operator that describes the wave propagation towards the detector up to the distance *z* [13]. Note that the complex differentiation is performed by AD in PyTorch employing intrinsically Wirtinger derivatives, similarly to other ptychography AD methods [119,120,123,135,136]. To do so, we wrote a small complex-algebra library [13], which can operate on a duplet datatype, where each 2D complex array is stored in its real and imaginary parts. To help the procedure correctly factorise the probe and the object, we included an L1 regularisation term, which takes into account energy conservation constraints: the object *x* cannot behave as a source, so the transmission function cannot present values larger than 1 in magnitude. $\left|\right|x_{x>1}{\left|\right|}_{1}$ indeed selectively penalises only the element with a magnitude greater than 1. The key element for such regularisation is the *fancy indexing* capability in PyTorch, inherited from Numpy.

The operator $D_{z}$ can be any single-pass wave-propagation routine; for the far-field case, the 2D Fourier Transform alone (implemented in PyTorch as a GPU function) reasonably approximates the field, as the ad hoc phase factors are automatically included in the probe *p* itself [4,103] during the reconstruction, thanks to the diversity in the dataset. In the case of near-field [137], the angular spectrum is a good compromise as it is relatively easy to implement, and it is also the most accurate solution [138]. To increase the execution speed, we cached the prefactors as much as possible [138], leaving only the Fourier Transform and the final element-wise multiplication to be calculated at run-time [13].

The model described so far can optimise the object *x* and the probe *p*, also in a multi-mode fashion [109]; to include the refinement of the prorogation distance *z*, we have to calculate more prefactors in $D_{z}$ at each iteration, making the algorithm inevitably slower.

In a typical ptychography code, the crop is simply obtained by slicing the array at a predefined set of coordinates and with fixed dimensions; this operation defines the so-called “computational box”. Slicing, however, is not differentiable as it involves integer quantities. Position refinement indeed requires a different type of crop operator, which can be emulated by an affine transform (which is differentiable). Such an operator exploits the same component described previously in the text and computes the 2D translation and rescale needed to produce the particular computational box at hand. More details on the generation of the affine transform matrix for each diffraction pattern can be found in [13,17]. Inevitably, the price to pay for the optimisation of new parameters is the use of a formalism that slows down the entire algorithm.

The final computational model is thus written as the composition of differentiable functions in the optimisable arrays $x,p,r_{xi},r_{yi},z$, which are the parameters appearing on the left-hand member of Equation (14).

## 3. Results

In this section, we present the reconstructions obtained for each of the aforementioned techniques. The CS and ptychography datasets were acquired during an experiment carried out at *TwinMic*, the soft-X-ray spectromicroscopy beamline [24,139] of the Elettra synchrotron facility. The sample is composed of a group of chemically fixed mesothelial cells (Mesenchymal–Epithelial Transition *Met5A*) grown on silicon nitride windows and exposed to asbestos fibres [13,24,140]. An energy of 1260 eV was used, paired with a secondary source [24] of 15 μm.

### 3.1. CS Reconstructions

Differently from the work reported in [25,26], here we show an application of CS for a fast STXM measurement performed by using a regular but sparse sampling mask. This kind of acquisition can be employed to further speed up the acquisition of the final XRF sampling mask, which typically depends upon an inspection of the STXM map (see Section 2.1). Such scan modality is compatible with the position control system of the microscope, as the input can be a list of sorted coordinates. The total scan area spans a range of 80 × 80 μm, for a total of 400 × 400 pixels, providing a pixel size of 200. Using such a large area and high-resolution setup requires employing a dwell time of 80 per scan point, totalling more than 4 h for an acquisition. By using CS, we can sample just 10% of the area (within one-tenth of the time) and recover the sample with a limited deterioration in quality.

**Figure 1:**
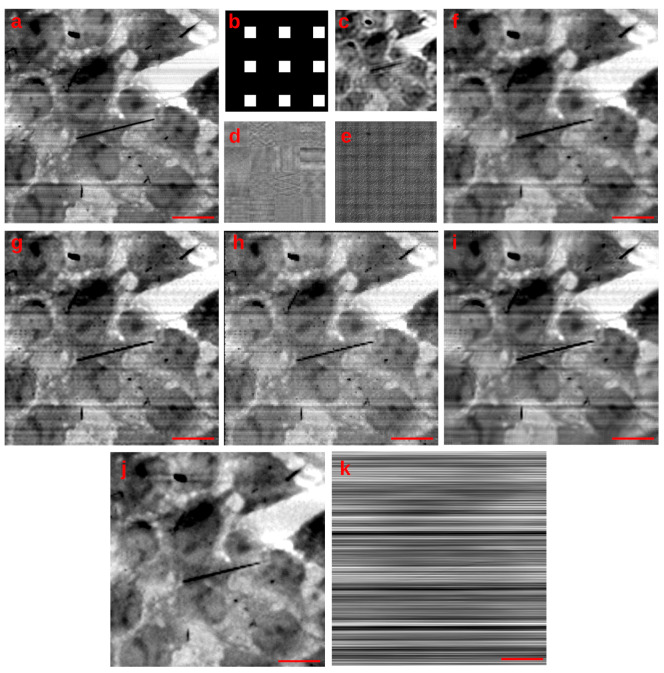
CS reconstruction with many algorithms. The red bar is 20 μm long. Panel (**a**): original full STXM image (ground truth) of the Met5A cells; panel (**b**): regular sensing mask; all the subsequent panels show the output of each CS algorithm applied on the same sparsely sampled image; panel (**c**):DR algorithm; panel (**d**): OMP algorithm; panel (**e**): NS algorithm; panel (**f**): proposed AD-based method; panel (**g**): Biharmonic inpainting; panel (**h**): Telea inpainting algorithm; panel (**i**): FSR inpainting algorithm; panel (**j**): proposed background-corrected output; panel (**k**): background estimation during the reconstruction of (**j**).

Figure 1 displays the results of many CS algorithms described in Section 1.4, compared to the proposed AD method: panel (a) shows the ground truth STXM acquired with a dense raster scan. From this image, we can realistically simulate the effects of sparse sampling by employing a regular sensing mask—a zoomed version is in Figure 1b—where only 10% of the pixel values are actually measured. Figure 1c shows the output of a Douglas–Rachford (DR) iterative thresholding algorithm; even after 1000 iterations, the final result is extremely blurred. While the use of a regular mask allows for a computationally simple interpolation scheme [26], it mines the applicability of a regular CS algorithm such as OMP (Section 1.4); the Restricted Isometry Property (RIP condition) indeed requires complete incoherence (randomness) between the sampling matrix and the underlying signal. When this condition is not met, the OMP algorithm stagnates, producing completely unusable results, such as the one in Figure 1d. A similar result can be observed in the output produced by the Naiver–Stokes (NS) inpainting algorithm; see Figure 1e.

**Figure 2:**
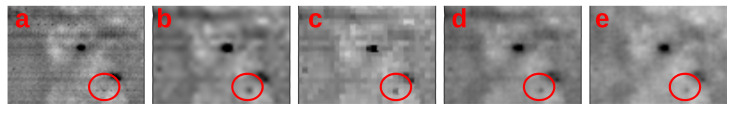
Detailed view of a zoomed area in Figure 1; ground truth (**a**), Biharmonic inpainting (**b**), Telea method (**c**); the proposed method provides a good reconstruction of the fine detail both in the uncorrected (panel **d**) and background corrected version (panel **e**).

Figure 1f shows the output of our proposed AD-based algorithm, which provides the least amount of artefacts, also compared to Biharmonic inpainting (panel g), Telea inpainting (panel h) and FSR inpainting (panel i). While the output of the Telea algorithm appears sharp, it presents a very blocky appearance. Nearest-neighbours-interpolation artefacts can also be spotted for panel (g), where small black features are grown with respect to the ground truth (see Figure 2). A very unnatural appearance with blurred borders can be seen in panel (i), where artefacts build up, especially in the external region.

The likelihood of having instabilities during an experiment increases with the time required for the data acquisition procedure. STXM and XRF experiments are often cursed by this (note the stripes in each panel of Figure 1). Within an AD framework, one can easily implement a new computational model, which takes into account the presence of a slowly varying function (beam intensity), which modulates the latent object in magnitude. The new computational model is presented in Equation (15):

(15) $$L\left(x,w\right)=||\Phi \Psi x-\frac{y}{\Phi w}{\left|\right|}_{2}^{2}+{\left||x|\right|}_{1}+V\left(w\right);$$

where the array *w* appears as a new trainable variable, with the same shape of *x*. In order to enforce a slow-varying behaviour that approximates slowly varying characteristics of the beam intensity, we apply a 1D version of the Total Variation regularisation V() on the serialised version of *w*. The output of this procedure is shown in Figure 1 panel (j), where the striping artefacts have been removed automatically *during the reconstruction* and not as the output of a post-processing filter; the estimated background, reshaped as *x* is shown in Figure 1k.

Figure 2 shows a detailed view of the CS reconstruction in Figure 1; note how the small circled dot in the ground truth (panel a) is blurred and increased in size for the Biharmonic and the Telea method (respectively panel b and c); the proposed method provides a good reconstruction of the fine detail both in the uncorrected (panel d) and background corrected version (panel e).

### 3.2. SISR Reconstructions

**Figure 3:**
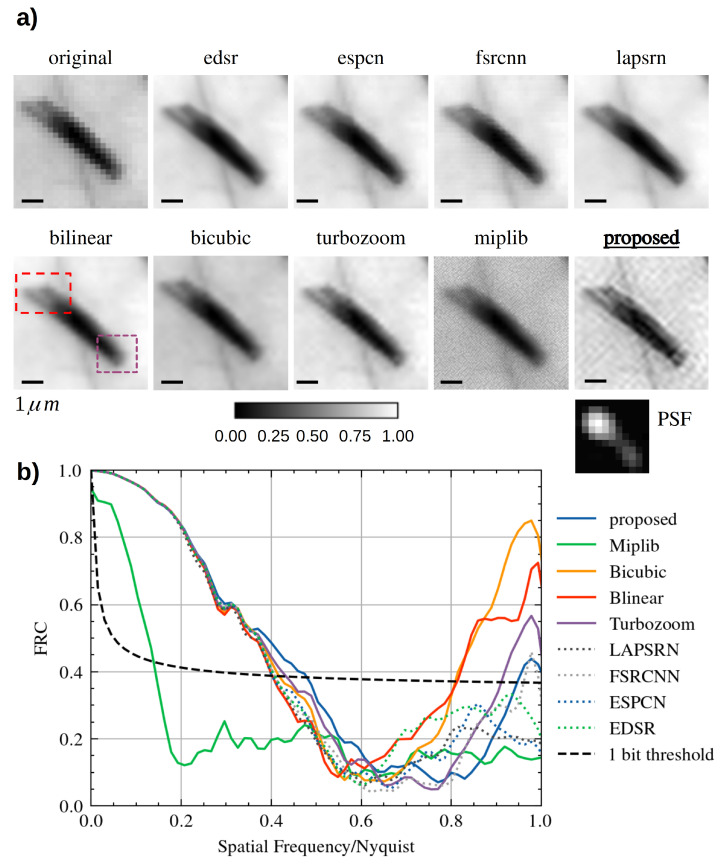
SISR reconstruction applied to a 26×26 pixel STXM scan. Panel (**a**): Compared to many 4× algorithms, the proposed method allows us to reconstruct the structure of each individual rod. The movement of the sample is described by a correctly estimated PSF; Panel (**b**) the Fourier Ring Correlation curves for each method in panel (**a**). The curve for the proposed method intercepts the 1 bit threshold (correlation factor of roughly 0.4) at the highest spatial frequency while maintaining a low amount of artefacts.

Single Image Super Resolution has been used to increase the level of detail of an STXM dataset, where the pixel size of 218 —fixed by the focusing characteristics of the FZP at 1260 eV—was not small enough to resolve the finer details. The sample is a different region of the *Met5A* sample, where many asbestos fibres aggregated, forming a cluster. Figure 3a shows the original STXM map of 26×26 pixels and the results of different SISR methods applied to that image; each recovered HR image has a resolution of 104×104 pixels (4× up-sample factor).

In Figure 3a, each reconstruction is normalised and shown at the same level of contrast. Neither the bilinear or the bicubic interpolation provide a good reconstruction: a bogus feature builds up, appearing as a “cross-like” artefact between the top leftmost fibres (red rectangle). A recent very fast reconstruction-based SISR method, “turbozoom” [46], is unfortunately tricked into generating the “cross-like” artefact and fails at recovering the correct rod profile. We also tried a blind deconvolution method (implemented in the “miplib” package [129]) applied on a bilinear interpolation, but no apparent increase in quality was observed.

On the contrary, the proposed method based on Equation (11) correctly separates the three rods in the top red rectangle, enhancing the individual contrast characteristics of each fibre in the cluster. This avoids the generation of the “cross-like” artefact. Furthermore, the end of the rods in the purple rectangle is better resolved. Moreover, during the STXM scan, a strong drift of the sample was observed, much bigger than the one scan step. The proposed method correctly disentangles this spurious movement from the object, estimating a Point Spread Function (PSF) that closely describes a motion blur.

SISR is currently receiving lots of attention from both the industry and the Image Processing research community, especially after the advent of Deep-Learning-based methods. Indeed, we also decided to test four different state-of-the-art Convolutional Neural Networks (CNN)—SISR techniques (ESDR [51], ESPCN [52], FSRCNN [53], and LAPSRN [54]). Being trained on natural images, these tested methods generalise poorly on X-ray microscopy images (see the blocking artefacts in FSRCNN, likely triggered by the intrinsic nature of the noise of a scanning technique). However, ESDR is somehow resolving the top-left features of the rods (red rectangle) while still blurring the bottom-right ends (purple rectangle).

The Fourier Ring Correlation [129] can be used to estimate the increase in resolution; for each tested method, the corresponding curve has been calculated and is plotted on the same graph of Figure 3b: as can be seen, the curve corresponding to the proposed AD-based reconstruction method (blue) intercepts the 1 bit threshold at the highest spatial frequency, meaning that at that spatial frequency, the signal-to-noise ratio is the highest. The FRC curves are not monotonic, but after roughly half of the Nyquist rate, many curves rise again: this behaviour can likely be explained by the build-up of high-frequency artefacts; among the tested method, the proposed algorithm (blue curve) provides the finest details, while limiting the artefacts components.

### 3.3. Micro/Nano—Tomography Reconstructions

**Figure 4:**
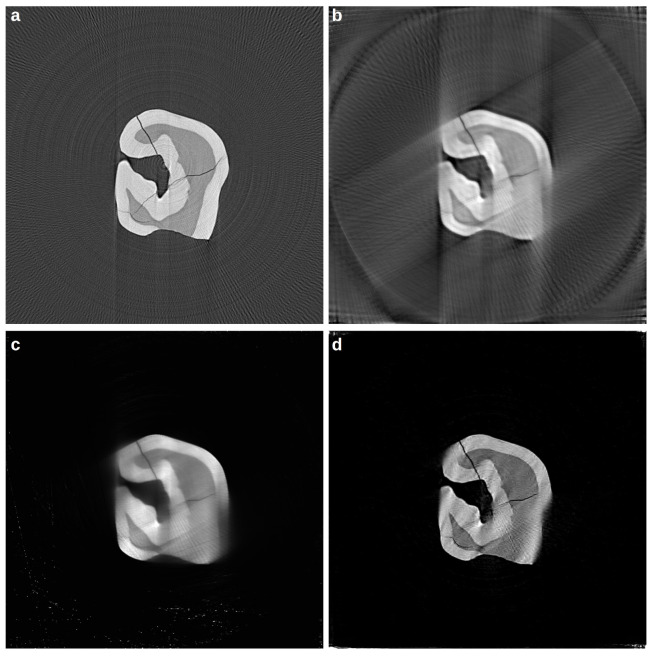
Micro-CT dataset reconstruction of a tooth, from the Tomopy software package [141]: panel (**a**): gridrec ground truth employing all the 180 projections (1° step). A simulated dataset is obtained by reducing the angle range to 120° and by increasing the angle step to 2° and is reconstructed with SIRT (panel **b**), MLEM (panel **c**), and the proposed AD-based method (panel **d**).

We tested our tomography reconstruction algorithm based on Equation (12) on two synchrotron radiation datasets and compared it against state-of-the art solutions (SIRT and MLEM, running on the GPU), implemented in the advanced TomoPy [141] software package. The first dataset is the “tooth” sinogram included in the TomoPy [141], also showcased in [142]. The original micro-CT dataset consists of 180 projections acquired with a step of 1°. The gridrec reconstruction of this dataset is shown in Figure 4a. To emulate a limited angle and sparsely acquired tomogram, we subsampled the dataset by a factor of two and reduced the rotation range to 120°, producing a resulting dataset of 60 projections. SIRT [72] (Figure 4b) and MLEM [71] (Figure 4c) were used to compare the performance of the proposed algorithm. To produce the reconstruction in panel (b), the SIRT algorithm was iterated for 500 iterations (20 s), but the algorithm reached a reasonably good convergence already after 20 iterations. The MLEM algorithm was used for 12 iterations (2 s); this number was chosen to reduce the number of artefacts in the reconstruction, which build up rapidly from iteration 13. The proposed method formalised in Equation (10) produced the reconstruction in panel (d), which appears sharp and with the highest degree of similarity to the ground truth. Note how the use of the CS-inspired minimisation in the transform domain (DWT) allowed for a good reconstruction of the global object shape; this can be seen especially at the border in the bottom-right part of the tooth, which was clearly in the unobserved angle range.

**Figure 5:**
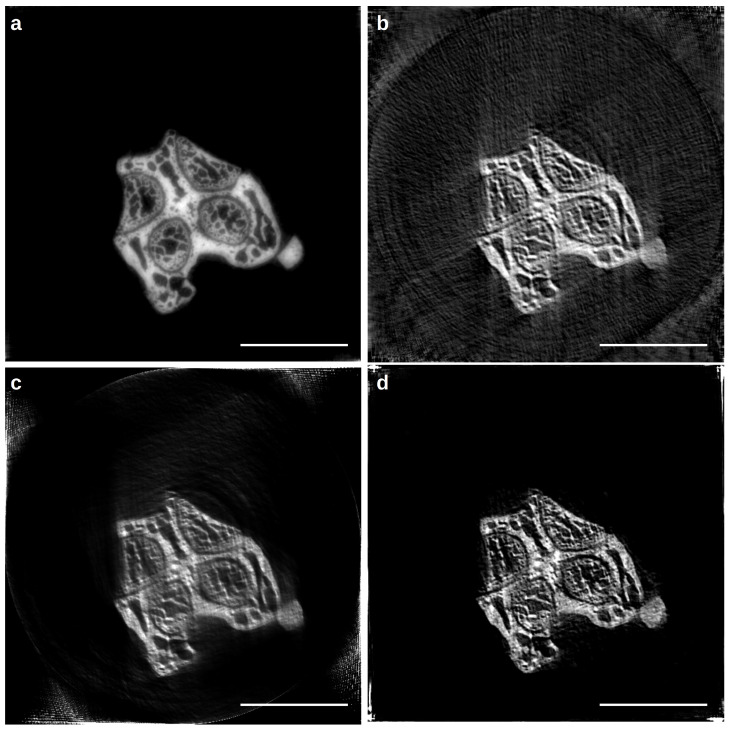
Micro-CT dataset reconstructions of a mouse femur [144]; panel (**a**) was obtained employing SIRT for 100 iterations, using all the available projections; a limited angle (120°) and sparse version (1.2° step) of the dataset was synthetically produced; the same dataset was reconstructed with SIRT (100 iterations—Panel **b**), MLEM (50 iterations, panel **c**) and the proposed AD-based method (panel **d**). The white bar is 1 mm long.

The second dataset is a different micro-CT scan performed at the *Syrmep* beamline [143] of the Elettra Synchrotron facility and used in [94,144]. A mouse femur [144] was acquired with an angle ranging between 0 and 180°, with steps of 0.4°, totalling 450 projections. The ground truth has been produced by using all the available projections with the SIRT algorithm, which was run for 100 iterations (Figure 5a). Similarly to the previous case, we simulated a sparsely sampled and limited-angle dataset by taking one projection of every three (step of 1.2°), again limiting the observations to 120°.

Figure 5b shows the output of the SIRT reconstruction algorithm, where the effects of the missing wedge problem are highly visible (smearing at the borders). MLEM (Figure 5c) produces an higher quality reconstruction compared to panel (b); similarly to the previous case, the proposed algorithm in panel (d) produces a reconstruction with crisper details and higher contrast.

**Figure 6:**
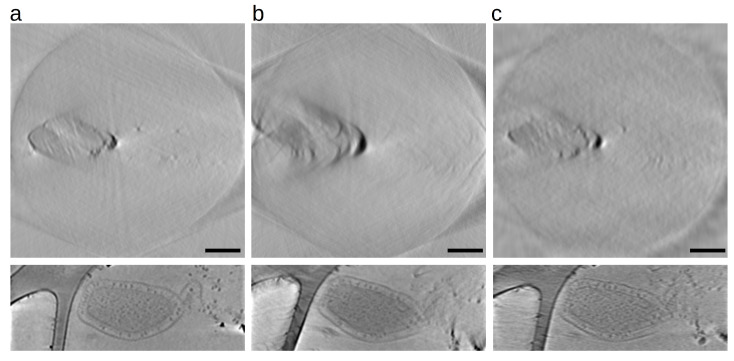
Cryo-nano-tomography reconstruction of the *Pyrodictium* dataset [80,145]; panel (**a**) reconstruction of the manually refined dataset using TomoJ; panel (**b**) automatic alignment with a simple correction model (detector shifts only [81]); panel (**c**) automatic alignment with the proposed simple correction model (detector shifts and angle) implemented in the AD framework. The black bar is 200 nm long.

To test the reconstruction method described by Equation (12), we used an electron tomography dataset published in [80,85] and well-known in the nanotomography community, being one of the tutorial datasets released for the Tomoj reconstruction software [145]. The *Pyrodictium abyssi cell strain TAG11* dataset is composed of 91 (512×512) projections, with a tilt step of 1.5°. The pixel size of each image is 3.24 nm. As previously described, limited angle and large misalignment are co-occurring in the same nanotomography experiment, producing a dataset that can not be reconstructed in a straightforward manner. Figure 6 shows different reconstructions obtained with three alignment/reconstruction methods, in both the axial and longitudinal plane. The black dots are nanobeads, which are typically added to the sample before the cryostate, in order to create a set of landmarks for the semi-automatic registration of the tilt series [75,80]. To reduce the computation time, each projection was rescaled to 256×256 pixel. Panel (a) displays the output of a manual alignment procedure carried out with Tomoj [80], where the automatically tracked landmarks were refined manually. This type of correction is considered the best we can obtain from this dataset; volume slices are well separated, and the gold nano-beads appear spherical in the axial plane. The aligned set of projections was processed by SIRT for 30 iterations.

Panel (b) instead displays the output of a fully automatic reconstruction procedure carried out by employing the joint alignment-reconstruction method described in [81,94] (for details, see Section 1.6): in this case, the correction model solely takes into account a set of shifts at the detector plane. The correction is indeed way simpler than the full 3D refinement model in [80], but it is the only algorithm of this type that is readily available in a CT reconstruction framework. SIRT was used as the base algorithm for the reconstruction, and in total, the optimisation routine iterated for 100 steps. In the axial plane, the beads appear elongated, which is a sign of an incomplete alignment.

Panel (c) shows the output of the proposed method (Equation (12)), which refines not only 2D shifts at the detector plane but also a detector tilt angle, which is common for all the projections. Compared to panel (b), the amount of misalignment is greatly reduced (see the axial plane). This kind of result can suffice for a preview, as the landmark detection and tracking alone (in the semi-automatic positions, refinement in panel (a)) takes the same time as the full reconstruction with the proposed method (5 min).

### 3.4. Ptychography Reconstructions

A different region of the *Met5A* sample has been observed through ptychography. The detector was placed in the far field $\left(z_{sd}=70\right)$, and the sample was illuminated with a curved wavefront produced by a defocused zone plate, with a focus-to-sample distance $z_{fs}$ of 350 μm. The dataset is composed of a set of 121 diffraction patterns acquired with a Princeton CCD camera, with a regular scan path [24]. We demonstrated [118] that during real experiments, we do not need to use special sampling schemes to avoid the raster grid pathology, as the mechanical position errors of the sample stage add a pseudo-random jitter on each position. Diffraction patterns are dark subtracted, centred and cropped to a set of (1024×1024) pixel images. The resulting pixel size is of roughly 36.

**Figure 7:**
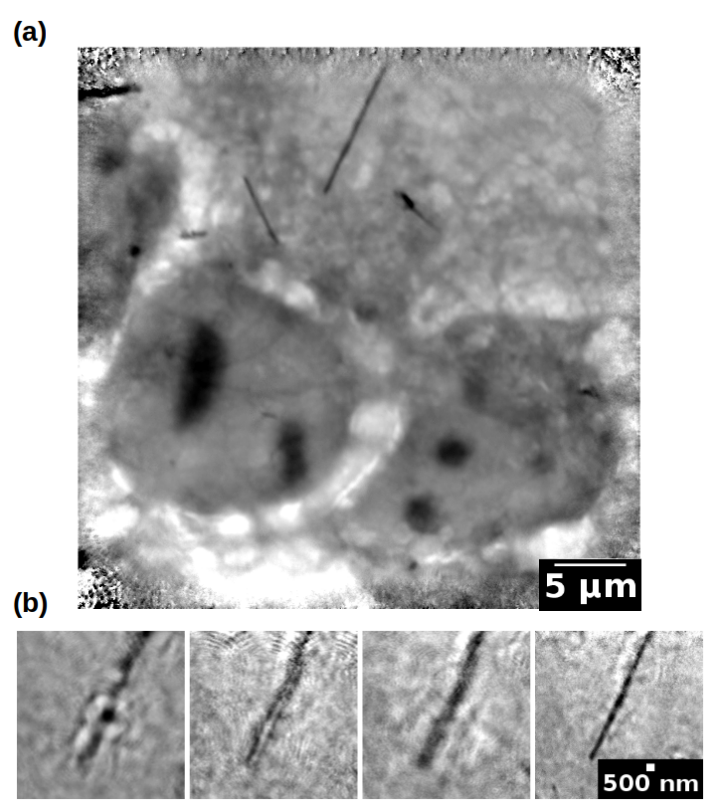
Ptychography reconstruction through AD [13]; panel (**a**): phase reconstruction of a Met5A sample; panel (**b**): comparison of different methods (see text) for the *same* propagation distance. Only the proposed method (last) correctly refines the parameters.

As already mentioned, the technique is very sensitive to geometrical parameter estimation. Indeed, the data analysis is typically performed by manually estimating the optical distances; a set of coarse reconstructions are performed by sweeping the parameters, then each reconstruction is carefully inspected to choose the best value. The positions vector is instead automatically refined [114,115,116,118]. Here we show the output of a ptychography reconstruction algorithm, which is automatically able to estimate the correct propagation distance [13]: Figure 7a shows the phase reconstruction of the sample. From a manually tuned virtual distance of 0.24, the method estimated instead a distance of 0.37. This final estimated value takes into account not only a mere distance correction but also positions and beam energy, as the exponential factor in the propagation operator is directly proportional to all these three parameters; this final value is the one corresponding to a minimum in the parameter-loss space. Figure 7b shows the effect of different reconstruction algorithms on a particular ROI of the sample. The algorithms involved are, respectively, DM [105], M-ePIE [102], M-rPIE [118] and, finally, the proposed AD-based algorithm [13].

## 4. Conclusions

In this manuscript, we described the results of an ongoing research on Automatic Differentiation methods applied to inverse microscopy problems. By realising that many computational imaging techniques can be solved by a common approach based on sampling, sparsity and error minimisation, we developed a modular framework that can be adapted to solve compressive sensing, super-resolution, tomography and ptychography. This allows the prototyping of ready-to-be-used algorithms in a fast manner, blurring out the border between the development and production phases. Fast prototyping allows us to experiment with multiple concepts across many fields and techniques. We showed how the proposed AD-based algorithms are not only easy to implement and reasonably fast but also provide comparable results, and in many cases superior to many state-of-the-art solutions. Prototypes for such reconstruction algorithms can be written in a reduced amount of time by non-experts and can also be adapted to parameter optimisation, mitigating various setup flaws. Even if an optimisation routine written by a software engineer will be the fastest solution, thanks to advanced GPU-enabled AD frameworks such as PyTorch the gap in speed can be underlooked if compared with the gain in reconstruction quality. If combined, these aspects make these methods available for a drop-in replacement within many analysis pipelines.

## Data Availability

Tomography dataset 1 is part of the Tomopy [141] software suite. Tomography dataset 2 was kindly donated by Dr. Francesco Brun and is available from [94]. Tomography dataset 3 has been used in [80] and is part of the Tomoj software suite tutorial [145]. The ptychography dataset/code is available from [13]. CS/SISR/CT code is available from [146].

## Abbreviations

The following abbreviations are used in this manuscript:

AD	Automatic Differentiation
CCD	Charge-Coupled Device
CNN	Convolutional Neural Network
CS	Compressive Sensing
CT	Computed Tomography
DCT	Discrete Cosine Transform
DWT	Discrete Wavelets Transform
DL	Deep Learning
FOV	Field Of View
FRC	Fourier Ring Correlation
FZP	Fresnel Zone Plate
GPU	Graphics Processing Unit
PSF	Point Spread Function
SISR	Single Image Super Resolution
STXM	Scanning Transmission X-ray Microscopy
XRF	X-ray Fluorescence

## References

[1] De Andrade V., Nikitin V., Wojcik M., Deriy A., Bean S., Shu D., Mooney T., Peterson K., Kc P., Li K. (2021). Fast X-ray Nanotomography with Sub-10 nm Resolution as a Powerful Imaging Tool for Nanotechnology and Energy Storage Applications. Adv. Mater..

[2] Candes E., Wakin M. (2008). An Introduction To Compressive Sampling. IEEE Signal Process. Mag..

[3] Chen H., He X., Qing L., Wu Y., Ren C., Sheriff R.E., Zhu C. (2022). Real-world single image super-resolution: A brief review. Inf. Fusion.

[4] Rodenburg J.M., Faulkner H.M. (2004). A phase retrieval algorithm for shifting illumination. Appl. Phys. Lett..

[5] McCloskey S. (2022). Computational Imaging. Adv. Comput. Vis. Pattern Recognit..

[6] Guizar-Sicairos M., Fienup J.R. (2008). Phase retrieval with transverse translation diversity: A nonlinear optimization approach. Opt. Express.

[7] Thibault P., Guizar-Sicairos M. (2012). Maximum-likelihood refinement for coherent diffractive imaging. New J. Phys..

[8] Donato S., Arana Peña L.M., Bonazza D., Formoso V., Longo R., Tromba G., Brombal L. (2022). Optimization of pixel size and propagation distance in X-ray phase-contrast virtual histology. J. Instrum..

[9] Brombal L., Arana Peña L.M., Arfelli F., Longo R., Brun F., Contillo A., Di Lillo F., Tromba G., Di Trapani V., Donato S. (2021). Motion artifacts assessment and correction using optical tracking in synchrotron radiation breast CT. Med. Phys..

[10] Bartholomew-Biggs M., Brown S., Christianson B., Dixon L. (2000). Automatic differentiation of algorithms. J. Comput. Appl. Math..

[11] Güneş Baydin A., Pearlmutter B.A., Andreyevich Radul A., Mark Siskind J. (2018). Automatic differentiation in machine learning: A survey. J. Mach. Learn. Res..

[12] Margossian C.C. (2019). A review of automatic differentiation and its efficient implementation. Wiley Interdiscip. Rev. Data Min. Knowl. Discov..

[13] Guzzi F., Kourousias G., Gianoncelli A., Billè F., Carrato S. (2021). A parameter refinement method for ptychography based on deep learning concepts. Condens. Matter.

[14] LeCun Y., Bengio Y., Hinton G. (2015). Deep learning. Nature.

[15] Li T.M., Gharbi M., Adams A., Durand F., Ragan-Kelley J. (2018). Differentiable programming for image processing and deep learning in halide. ACM Trans. Graph..

[16] Rios L.M., Sahinidis N.V. (2013). Derivative-free optimization: A review of algorithms and comparison of software implementations. J. Glob. Optim..

[17] Guzzi F., De Bortoli L., Molina R.S., Marsi S., Carrato S., Ramponi G. (2020). Distillation of an end-to-end oracle for face verification and recognition sensors. Sensors.

[18] Laue S. (2019). On the Equivalence of Forward Mode Automatic Differentiation and Symbolic Differentiation. http://xxx.lanl.gov/abs/1904.02990.

[19] Paszke A., Gross S., Massa F., Lerer A., Bradbury J., Chanan G., Killeen T., Lin Z., Gimelshein N., Antiga L. (2019). PyTorch: An imperative style, high-performance deep learning library. Proceedings of the 33rd International Conference on Neural Information Processing Systems, Vancouver, BC, Canada, 8–14 December 2019.

[20] Liu D.C., Nocedal J. (1989). On the limited memory BFGS method for large scale optimization. Math. Program..

[21] Andrew G., Gao J. (2007). Scalable training of L1-regularized log-linear models. Proceedings of the ACM International Conference Proceeding Series.

[22] Baraniuk R.G. (2007). Compressive Sensing [Lecture Notes]. IEEE Signal Process. Mag..

[23] Zhang J., Zhao C., Zhao D., Gao W. (2014). Image compressive sensing recovery using adaptively learned sparsifying basis via L0 minimization. Signal Process..

[24] Gianoncelli A., Bonanni V., Gariani G., Guzzi F., Pascolo L., Borghes R., Billè F., Kourousias G. (2021). Soft x-ray microscopy techniques for medical and biological imaging at twinmic–elettra. Appl. Sci..

[25] Kourousias G., Billè F., Borghes R., Alborini A., Sala S., Alberti R., Gianoncelli A. (2020). Compressive Sensing for Dynamic XRF Scanning. Sci. Rep..

[26] Kourousias G., Billè F., Borghes R., Pascolo L., Gianoncelli A. (2021). Megapixel scanning transmission soft X-ray microscopy imaging coupled with compressive sensing X-ray fluorescence for fast investigation of large biological tissues. Analyst.

[27] Vetal A.P., Singh D., Singh R.K., Mishra D. (2018). Reconstruction of apertured Fourier Transform Hologram using compressed sensing. Opt. Lasers Eng..

[28] Orović I., Papić V., Ioana C., Li X., Stanković S. (2016). Compressive Sensing in Signal Processing: Algorithms and Transform Domain Formulations. Math. Probl. Eng..

[29] Pilastri A.L., Tavares J.M.R. Reconstruction algorithms in compressive sensing: An overview. Proceedings of the 11th Edition of the Doctoral Symposium in Informatics Engineering (DSIE-16).

[30] Peyré G. (2011). The numerical tours of signal processing part 2: Multiscale processings. Comput. Sci. Eng..

[31] Li S., Qi H. (2015). A Douglas-Rachford Splitting Approach to Compressed Sensing Image Recovery Using Low-Rank Regularization. IEEE Trans. Image Process..

[32] Mallat S.G., Zhang Z. (1993). Matching Pursuits With Time-Frequency Dictionaries. IEEE Trans. Signal Process..

[33] Cai T.T., Wang L. (2011). Orthogonal matching pursuit for sparse signal recovery with noise. IEEE Trans. Inf. Theory.

[34] Zhu H., Chen W., Wu Y. (2020). Efficient implementations for orthogonal matching pursuit. Electronics.

[35] Damelin S.B., Hoang N.S. (2018). On Surface Completion and Image Inpainting by Biharmonic Functions: Numerical Aspects. Int. J. Math. Math. Sci..

[36] Telea A. (2004). An Image Inpainting Technique Based on the Fast Marching Method. J. Graph. Tools.

[37] Bertalmío M., Bertozzi A.L., Sapiro G. Navier-Stokes, fluid dynamics, and image and video inpainting. Proceedings of the IEEE Computer Society Conference on Computer Vision and Pattern Recognition.

[38] Genser N., Seiler J., Schilling F., Kaup A. Signal and Loss Geometry Aware Frequency Selective Extrapolation for Error Concealment. Proceedings of the 2018 Picture Coding Symposium, PCS 2018—Proceedings.

[39] Seiler J., Kaup A. (2010). Complex-valued frequency selective extrapolation for fast image and video signal extrapolation. IEEE Signal Process. Lett..

[40] Wang Z., Chen J., Hoi S. (2021). Deep Learning for Image Super-Resolution: A Survey. Inf. Fusion.

[41] Guarnieri G., Fontani M., Guzzi F., Carrato S., Jerian M. (2021). Perspective registration and multi-frame super-resolution of license plates in surveillance videos. Forensic Sci. Int. Digit. Investig..

[42] Lehtinen J., Munkberg J., Hasselgren J., Laine S., Karras T., Aittala M., Aila T., Dy J., Krause A. (2018). Noise2Noise: Learning image restoration without clean data. Proceedings of the 35th International Conference on Machine Learning, ICML 2018, Stockholm, Sweden, 10–15 July 2018.

[43] Vicente A.N., Pedrini H. A learning-based single-image super-resolution method for very low quality license plate images. Proceedings of the 2016 IEEE International Conference on Systems, Man, and Cybernetics, SMC 2016—Conference Proceedings.

[44] Papyan V., Elad M. (2016). Multi-Scale Patch-Based Image Restoration. IEEE Trans. Image Process..

[45] Brifman A., Romano Y., Elad M. Turning a denoiser into a super-resolver using plug and play priors. Proceedings of the International Conference on Image Processing, ICIP.

[46] Eilers P.H., Ruckebusch C. (2022). Fast and simple super-resolution with single images. Sci. Rep..

[47] Zeyde R., Elad M., Protter M., Boissonnat J.D., Chenin P., Cohen A., Gout C., Lyche T., Mazure M.L., Schumaker L.L. (2012). On single image scale-up using sparse-representations. Lecture Notes in Computer Science (Including Subseries Lecture Notes in Artificial Intelligence and Lecture Notes in Bioinformatics).

[48] Sen P., Darabi S. Compressive image super-resolution. Proceedings of the Conference Record—Asilomar Conference on Signals, Systems and Computers.

[49] Freeman W.T., Jones T.R., Pasztor E.C. (2002). Example-based super-resolution. IEEE Comput. Graph. Appl..

[50] Glasner D., Bagon S., Irani M. Super-resolution from a single image. Proceedings of the IEEE International Conference on Computer Vision.

[51] Lim B., Son S., Kim H., Nah S., Lee K.M. (2017). Enhanced Deep Residual Networks for Single Image Super-Resolution. Proceedings of the IEEE Computer Society Conference on Computer Vision and Pattern Recognition Workshops, Honolulu, HI, USA, 21–26 July 2017.

[52] Shi W., Caballero J., Huszar F., Totz J., Aitken A.P., Bishop R., Rueckert D., Wang Z. (2016). Real-Time Single Image and Video Super-Resolution Using an Efficient Sub-Pixel Convolutional Neural Network. Proceedings of the IEEE Computer Society Conference on Computer Vision and Pattern Recognition, Las Vegas, NV, USA, 27–30 June 2016.

[53] Dong C., Loy C.C., Tang X., Leibe B., Matas J., Sebe N., Welling M. (2016). Accelerating the super-resolution convolutional neural network. Lecture Notes in Computer Science (Including Subseries Lecture Notes in Artificial Intelligence and Lecture Notes in Bioinformatics).

[54] Lai W.S., Huang J.B., Ahuja N., Yang M.H. (2017). Deep laplacian pyramid networks for fast and accurate super-resolution. Proceedings of the 30th IEEE Conference on Computer Vision and Pattern Recognition, CVPR 2017, Honolulu, HI, USA, 21–26 July 2017.

[55] Bruno P., Calimeri F., Marte C., Manna M., Moschoyiannis S., Peñaloza R., Vanthienen J., Soylu A., Roman D. (2021). Combining Deep Learning and ASP-Based Models for the Semantic Segmentation of Medical Images. Lecture Notes in Computer Science (Including Subseries Lecture Notes in Artificial Intelligence and Lecture Notes in Bioinformatics).

[56] Vo T.H., Nguyen N.T.K., Kha Q.H., Le N.Q.K. (2022). On the road to explainable AI in drug-drug interactions prediction: A systematic review. Comput. Struct. Biotechnol. J..

[57] Murdoch W.J., Singh C., Kumbier K., Abbasi-Asl R., Yu B. (2019). Definitions, methods, and applications in interpretable machine learning. Proc. Natl. Acad. Sci. USA.

[58] Yang X. (2020). An Overview of the Attention Mechanisms in Computer Vision. J. Phys. Conf. Ser..

[59] Hounsfield G.N. (1973). Computerized transverse axial scanning (tomography): I. Description of system. Br. J. Radiol..

[60] Szczykutowicz T.P., Toia G.V., Dhanantwari A., Nett B. (2022). A Review of Deep Learning CT Reconstruction: Concepts, Limitations, and Promise in Clinical Practice. Curr. Radiol. Rep..

[61] Pereiro E., Nicolás J., Ferrer S., Howells M.R. (2009). A soft X-ray beamline for transmission X-ray microscopy at ALBA. J. Synchrotron Radiat..

[62] Withers P.J., Bouman C., Carmignato S., Cnudde V., Grimaldi D., Hagen C.K., Maire E., Manley M., Du Plessis A., Stock S. (2021). X-ray computed tomography. Nat. Rev. Methods Prim..

[63] Morgan K.S., Siu K.K., Paganin D.M. (2010). The projection approximation versus an exact solution for X-ray phase contrast imaging, with a plane wave scattered by a dielectric cylinder. Opt. Commun..

[64] Soleimani M., Pengpen T. (2015). Introduction: A brief overview of iterative algorithms in X-ray computed tomography. Philos. Trans. R. Soc. A Math. Phys. Eng. Sci..

[65] Jacobsen C. (2018). Relaxation of the Crowther criterion in multislice tomography. Opt. Lett..

[66] Dowd B.A., Campbell G.H., Marr R.B., Nagarkar V.V., Tipnis S.V., Axe L., Siddons D.P. (1999). Developments in synchrotron x-ray computed microtomography at the National Synchrotron Light Source. Dev. X-ray Tomogr. II.

[67] Gordon R., Bender R., Herman G.T. (1970). Algebraic Reconstruction Techniques (ART) for three-dimensional electron microscopy and X-ray photography. J. Theor. Biol..

[68] Gianoncelli A., Vaccari L., Kourousias G., Cassese D., Bedolla D.E., Kenig S., Storici P., Lazzarino M., Kiskinova M. (2015). Soft X-Ray Microscopy Radiation Damage On Fixed Cells Investigated With Synchrotron Radiation FTIR Microscopy. Sci. Rep..

[69] Frachon T., Weber L., Hesse B., Rit S., Dong P., Olivier C., Peyrin F., Langer M. (2015). Dose fractionation in synchrotron radiation x-ray phase micro-tomography. Phys. Med. Biol..

[70] Mori I., Machida Y., Osanai M., Iinuma K. (2013). Photon starvation artifacts of X-ray CT: Their true cause and a solution. Radiol. Phys. Technol..

[71] Dempster A.P., Laird N.M., Rubin D.B. (1977). Maximum Likelihood from Incomplete Data Via the EM Algorithm. J. R. Stat. Soc. Ser. B (Methodol.).

[72] van der Sluis A., van der Vorst H.A. (1990). SIRT- and CG-type methods for the iterative solution of sparse linear least-squares problems. Linear Algebra Its Appl..

[73] Gregor J., Benson T. (2008). Computational analysis and improvement of SIRT. IEEE Trans. Med. Imaging.

[74] Kupsch A., Lange A., Hentschel M.P., Lück S., Schmidt V., Grothausmann R., Hilger A., Manke I. (2016). Missing wedge computed tomography by iterative algorithm DIRECTT. J. Microsc..

[75] Sorrentino A., Nicolás J., Valcárcel R., Chichón F.J., Rosanes M., Avila J., Tkachuk A., Irwin J., Ferrer S., Pereiro E. (2015). MISTRAL: A transmission soft X-ray microscopy beamline for cryo nano-tomography of biological samples and magnetic domains imaging. J. Synchrotron Radiat..

[76] Guay M.D., Czaja W., Aronova M.A., Leapman R.D. (2016). Compressed sensing electron tomography for determining biological structure. Sci. Rep..

[77] Moebel E., Kervrann C. (2020). A Monte Carlo framework for missing wedge restoration and noise removal in cryo-electron tomography. J. Struct. Biol. X.

[78] Xu J., Mahesh M., Tsui B.M. (2009). Is Iterative Reconstruction Ready for MDCT?. J. Am. Coll. Radiol..

[79] Ding G., Liu Y., Zhang R., Xin H.L. (2019). A joint deep learning model to recover information and reduce artifacts in missing-wedge sinograms for electron tomography and beyond. Sci. Rep..

[80] Sorzano C.O.S., Messaoudi C., Eibauer M., Bilbao-Castro J.R., Hegerl R., Nickell S., Marco S., Carazo J.M. (2009). Marker-free image registration of electron tomography tilt-series. BMC Bioinform..

[81] Gürsoy D., Hong Y.P., He K., Hujsak K., Yoo S., Chen S., Li Y., Ge M., Miller L.M., Chu Y.S. (2017). Rapid alignment of nanotomography data using joint iterative reconstruction and reprojection. Sci. Rep..

[82] Bogensperger L., Kobler E., Pernitsch D., Kotzbeck P., Pieber T.R., Pock T., Kolb D. (2022). A joint alignment and reconstruction algorithm for electron tomography to visualize in-depth cell-to-cell interactions. Histochem. Cell Biol..

[83] Frank J., McEwen B.F., Frank J. (1992). Alignment by Cross-Correlation. Electron Tomography.

[84] Kremer J.R., Mastronarde D.N., McIntosh J.R. (1996). Computer visualization of three-dimensional image data using IMOD. J. Struct. Biol..

[85] Sorzano C.O., de Isidro-Gómez F., Fernández-Giménez E., Herreros D., Marco S., Carazo J.M., Messaoudi C. (2020). Improvements on marker-free images alignment for electron tomography. J. Struct. Biol. X.

[86] Yu H., Xia S., Wei C., Mao Y., Larsson D., Xiao X., Pianetta P., Yu Y.S., Liu Y. (2018). Automatic projection image registration for nanoscale X-ray tomographic reconstruction. J. Synchrotron Radiat..

[87] Zhang J., Hu J., Jiang Z., Zhang K., Liu P., Wang C., Yuan Q., Pianetta P., Liu Y. (2021). Automatic 3D image registration for nano-resolution chemical mapping using synchrotron spectro-tomography. J. Synchrotron Radiat..

[88] Jun K., Yoon S. (2017). Alignment Solution for CT Image Reconstruction using Fixed Point and Virtual Rotation Axis. Sci. Rep..

[89] Han R., Wang L., Liu Z., Sun F., Zhang F. (2015). A novel fully automatic scheme for fiducial marker-based alignment in electron tomography. J. Struct. Biol..

[90] Han R., Wan X., Wang Z., Hao Y., Zhang J., Chen Y., Gao X., Liu Z., Ren F., Sun F. (2017). AuTom: A novel automatic platform for electron tomography reconstruction. J. Struct. Biol..

[91] Woolcot T., Kousi E., Wells E., Aitken K., Taylor H., Schmidt M.A. (2018). An evaluation of systematic errors on marker-based registration of computed tomography and magnetic resonance images of the liver. Phys. Imaging Radiat. Oncol..

[92] Han R., Li G., Gao X. (2021). Robust and ultrafast fiducial marker correspondence in electron tomography by a two-stage algorithm considering local constraints. Bioinformatics.

[93] Han R., Bao Z., Zeng X., Niu T., Zhang F., Xu M., Gao X. (2019). A joint method for marker-free alignment of tilt series in electron tomography. Bioinformatics.

[94] Guzzi F., Kourousias G., Gianoncelli A., Pascolo L., Sorrentino A., Billè F., Carrato S. (2021). Improving a rapid alignment method of tomography projections by a parallel approach. Appl. Sci..

[95] Di Z.W., Chen S., Gursoy D., Paunesku T., Leyffer S., Wild S.M., Vogt S. (2019). Optimization-based simultaneous alignment and reconstruction in multi-element tomography. Opt. Lett..

[96] Odstrčil M., Holler M., Raabe J., Guizar-Sicairos M. (2019). Alignment methods for nanotomography with deep subpixel accuracy. Opt. Express.

[97] Guizar-Sicairos M., Thibault P. (2021). Ptychography: A solution to the phase problem. Phys. Today.

[98] Pfeiffer F. (2018). X-ray ptychography. Nat. Photonics.

[99] Paganin D., Gureyev T.E., Mayo S.C., Stevenson A.W., Nesterets Y.I., Wilkins S.W. (2004). X-ray omni microscopy. J. Microsc..

[100] Abbey B., Nugent K.A., Williams G.J., Clark J.N., Peele A.G., Pfeifer M.A., De Jonge M., McNulty I. (2008). Keyhole coherent diffractive imaging. Nat. Phys..

[101] Rodenburg J. (1992). The theory of super-resolution electron microscopy via Wigner-distribution deconvolution. Philos. Trans. R. Soc. Lond. Ser. A Phys. Eng. Sci..

[102] Maiden A.M., Rodenburg J.M. (2009). An improved ptychographical phase retrieval algorithm for diffractive imaging. Ultramicroscopy.

[103] Maiden A., Johnson D., Li P. (2017). Further improvements to the ptychographical iterative engine. Optica.

[104] Marchesini S., Tu Y.C., Wu H.T. (2016). Alternating projection, ptychographic imaging and phase synchronization. Appl. Comput. Harmon. Anal..

[105] Thibault P., Dierolf M., Menzel A., Bunk O., David C., Pfeiffer F. (2008). High-Resolution Scanning X-ray Diffraction Microscopy. Science.

[106] Pelz P.M., Qiu W.X., Bücker R., Kassier G., Miller R.J.D. (2017). Low-dose cryo electron ptychography via non-convex Bayesian optimization. Sci. Rep..

[107] Spence J., Weierstall U., Howells M. (2004). Coherence and sampling requirements for diffractive imaging. Ultramicroscopy.

[108] Vartanyants I., Robinson I. (2003). Origins of decoherence in coherent X-ray diffraction experiments. Opt. Commun..

[109] Thibault P., Menzel A. (2013). Reconstructing state mixtures from diffraction measurements. Nature.

[110] Li P., Batey D.J., Edo T.B., Parsons A.D., Rau C., Rodenburg J.M. (2016). Multiple mode x-ray ptychography using a lens and a fixed diffuser optic. J. Opt..

[111] Shi X., Burdet N., Batey D., Robinson I. (2018). Multi-Modal Ptychography: Recent Developments and Applications. Appl. Sci..

[112] Xu W., Ning S., Zhang F. (2021). Numerical and experimental study of partial coherence for near-field and far-field ptychography. Opt. Express.

[113] Maiden A., Humphry M., Sarahan M., Kraus B., Rodenburg J. (2012). An annealing algorithm to correct positioning errors in ptychography. Ultramicroscopy.

[114] Zhang F., Peterson I., Vila-Comamala J., Diaz A., Berenguer F., Bean R., Chen B., Menzel A., Robinson I.K., Rodenburg J.M. (2013). Translation position determination in ptychographic coherent diffraction imaging. Opt. Express.

[115] Mandula O., Elzo Aizarna M., Eymery J., Burghammer M., Favre-Nicolin V. (2016). PyNX.Ptycho: A computing library for X-ray coherent diffraction imaging of nanostructures. J. Appl. Crystallogr..

[116] Guzzi F., Kourousias G., Billè F., Pugliese R., Reis C., Gianoncelli A., Carrato S. (2018). Refining scan positions in Ptychography through error minimisation and potential application of Machine Learning. J. Instrum..

[117] Dwivedi P., Konijnenberg A.P., Pereira S.F., Meisner J., Urbach H.P. (2019). Position correction in ptychography using hybrid input–output (HIO) and cross-correlation. J. Opt..

[118] Guzzi F., Kourousias G., Billè F., Pugliese R., Gianoncelli A., Carrato S. (2022). A modular software framework for the design and implementation ofptychography algorithms. PeerJ Comput. Sci..

[119] Du M., Kandel S., Deng J., Huang X., Demortiere A., Nguyen T.T., Tucoulou R., De Andrade V., Jin Q., Jacobsen C. (2021). Adorym: A multi-platform generic X-ray image reconstruction framework based on automatic differentiation. Opt. Express.

[120] Du M., Nashed Y.S., Kandel S., Gürsoy D., Jacobsen C. (2020). Three dimensions, two microscopes, one code: Automatic differentiation for x-ray nanotomography beyond the depth of focus limit. Sci. Adv..

[121] Shenfield A., Rodenburg J.M. (2011). Evolutionary determination of experimental parameters for ptychographical imaging. J. Appl. Phys..

[122] Loetgering L., Rose M., Keskinbora K., Baluktsian M., Dogan G., Sanli U., Bykova I., Weigand M., Schütz G., Wilhein T. (2018). Correction of axial position uncertainty and systematic detector errors in ptychographic diffraction imaging. Opt. Eng..

[123] Loetgering L., Du M., Eikema K.S.E., Witte S. (2020). zPIE: An autofocusing algorithm for ptychography. Opt. Lett..

[124] Guzzi F., Kourousias G., Billè F., Pugliese R., Gianoncelli A., Carrato S. A Deep Prior Method for Fourier Ptychography Microscopy. Proceedings of the 2021 44th International Convention on Information, Communication and Electronic Technology (MIPRO).

[125] Harris C.R., Millman K.J., van der Walt S.J., Gommers R., Virtanen P., Cournapeau D., Wieser E., Taylor J., Berg S., Smith N.J. (2020). Array programming with NumPy. Nature.

[126] Virtanen P., Gommers R., Oliphant T.E., Haberland M., Reddy T., Cournapeau D., Burovski E., Peterson P., Weckesser W., Bright J. (2020). SciPy 1.0: Fundamental Algorithms for Scientific Computing in Python. Nat. Methods.

[127] Hu Z. (2018). Pytorch-DCT. https://github.com/zh217/torch-dct.

[128] Feinman R. (2021). Pytorch-Minimize. https://github.com/rfeinman/pytorch-minimize.

[129] Koho S., Tortarolo G., Castello M., Deguchi T., Diaspro A., Vicidomini G. (2019). Fourier ring correlation simplifies image restoration in fluorescence microscopy. Nat. Commun..

[130] Huber P.J. (1964). Robust Estimation of a Location Parameter. Ann. Math. Stat..

[131] Chambolle A., Duval V., Peyré G., Poon C. (2016). Geometric properties of solutions to the total variation denoising problem. Inverse Probl..

[132] Cotter F. (2020). Uses of Complex Wavelets in Deep Convolutional Neural Networks.

[133] (1999). A Wavelet Tour of Signal Processing.

[134] E. Riba D., Mishkin D.P.E.R., Bradski G. (2020). Kornia: An Open Source Differentiable Computer Vision Library for PyTorch. Proceedings of the Winter Conference on Applications of Computer Vision.

[135] Nashed Y.S., Peterka T., Deng J., Jacobsen C. (2017). Distributed Automatic Differentiation for Ptychography. Procedia Comput. Sci..

[136] Kandel S., Maddali S., Allain M., Hruszkewycz S.O., Jacobsen C., Nashed Y.S.G. (2019). Using automatic differentiation as a general framework for ptychographic reconstruction. Opt. Express.

[137] Stockmar M., Cloetens P., Zanette I., Enders B., Dierolf M., Pfeiffer F., Thibault P. (2013). Near-field ptychography: Phase retrieval for inline holography using a structured illumination. Sci. Rep..

[138] Paganin D.M. (2013). Coherent X-ray Optics.

[139] Gianoncelli A., Kourousias G., Merolle L., Altissimo M., Bianco A. (2016). Current status of the TwinMic beamline at Elettra: A soft X-ray transmission and emission microscopy station. J. Synchrotron Radiat..

[140] Cammisuli F., Giordani S., Gianoncelli A., Rizzardi C., Radillo L., Zweyer M., Da Ros T., Salomé M., Melato M., Pascolo L. (2018). Iron-related toxicity of single-walled carbon nanotubes and crocidolite fibres in human mesothelial cells investigated by Synchrotron XRF microscopy. Sci. Rep..

[141] Gürsoy D., De Carlo F., Xiao X., Jacobsen C. (2014). TomoPy: A framework for the analysis of synchrotron tomographic data. J. Synchrotron Radiat..

[142] Pouchard L., Juhas P., Park G., Dam H.V., Campbell S.I., Stavitski E., Billinge S., Wright C.J. (2020). Provenance Infrastructure for Multi-modal X-ray Experiments and Reproducible Analysis. Handbook on Big Data and Machine Learning in the Physical Sciences.

[143] Dullin C., di Lillo F., Svetlove A., Albers J., Wagner W., Markus A., Sodini N., Dreossi D., Alves F., Tromba G. (2021). Multiscale biomedical imaging at the SYRMEP beamline of Elettra—Closing the gap between preclinical research and patient applications. Phys. Open.

[144] Tavella S., Ruggiu A., Giuliani A., Brun F., Canciani B., Manescu A., Marozzi K., Cilli M., Costa D., Liu Y. (2012). Bone Turnover in Wild Type and Pleiotrophin-Transgenic Mice Housed for Three Months in the International Space Station (ISS). PLoS ONE.

[145] MessaoudiI C., Boudier T., Sorzano C.O.S., Marco S. (2007). TomoJ: Tomography software for three-dimensional reconstruction in transmission electron microscopy. BMC Bioinform..

[146] Guzzi F., Bille‘ F., Carrato S., Gianoncelli A. Kourousias, G Automatic Differentiation Methods for Computational Microscopy Experiments—Code. 2023. https://vuo.elettra.eu/pls/vuo/open_access_data_portal.show_view_investigation?FRM_ID=10664.

